# Assessment of Foot-and-Mouth Disease Trends in Türkiye Between 2005 and 2025

**DOI:** 10.1155/tbed/2756250

**Published:** 2025-11-16

**Authors:** Mafalda Pedro Mil-Homens, Margarida Arede, Daniel Beltrán-Alcrudo, Mark Hovari, Eran Raizman, Alberto Allepuz Palau

**Affiliations:** ^1^Departament de Sanitat I d'Anatomia Animals, Universitat Autònoma de Barcelona, Barcelona, Spain; ^2^State Institute for Health II, Task Force for Infectious Diseases (GI), Bavarian Health and Food Safety Authority (LGL), Munich, Germany; ^3^Food and Agriculture Organization of the United Nations (FAO), Regional Office for Europe and Central Asia, Budapest, Hungary

**Keywords:** FMD serotypes, FMD trends, geographical distribution, seasonality, time series

## Abstract

Foot-and-mouth disease (FMD) is a highly contagious viral illness that continues to threaten livestock health, productivity, and trade, particularly in countries like Türkiye where multiple FMD serotypes cocirculate. This study aimed to analyze the temporal and spatial distributions, serotype dynamics, and seasonal patterns of FMD in Türkiye between 2005 and 2025. The geographical distribution of national FMD surveillance data spanning 2005–2025 was analyzed, together with a time series analysis to evaluate trends and seasonality in the number of FMD-reported outbreaks (number of villages affected by FMD outbreaks). Additionally, event-driven outbreaks were assessed by analyzing the number of reported outbreaks when a serotype introduction occurred and during Kurban Bayramı festivities. The results showed that the number of FMD-reported outbreaks peaked between 2010 and 2016, with serotype O accounting for the majority of reported outbreaks. A decline in outbreaks followed, likely attributable to expanded vaccination coverage, improved diagnostic capacity, and the implementation of targeted control measures. Seasonal patterns indicated a higher concentration of outbreaks during the spring months. Furthermore, it was noted that introductions of serotypes, lineages, or sublineages contributed to an increase in outbreaks in the months surrounding these events, and the same was observed when Kurban Bayramı festivities occurred. Although Türkiye has made progress in reducing the FMD burden, the emergence of new serotypes highlights the ongoing risk of serotype diversification and underscores the need for adaptable, serotype-specific surveillance, and control strategies. Strengthening early detection systems, maintaining high vaccine coverage, and fostering regional cooperation remain essential for sustainable FMD management.

## 1. Introduction

Foot-and-mouth disease (FMD) is a highly contagious viral illness affecting cloven-hoofed animals, including cattle, pigs, sheep, and goats. Caused by the FMD virus (FMDV)—a single-stranded RNA virus from the genus *Aphthovirus* (family *Picornaviridae*)—the disease is classified into seven immunologically distinct serotypes: A, O, C, Asia 1, and South African Territories (SAT) 1, 2, and 3, each with multiple subtypes [1, 2]. Although FMD is rarely fatal in adult animals, it causes severe productivity losses due to reduced milk yield, weight loss, abortions, and lameness. It also restricts access to international markets, leading to significant economic repercussions [1, 3–5].

Globally, FMD remains a major transboundary animal disease with both endemic and epidemic profiles. Historic outbreaks, such as the 1997 epidemic in Taiwan and the 2001 crisis in the United Kingdom, demonstrate their capacity to cause widespread economic disruption, with losses estimated at billions of US dollars [6, 7]. In Türkiye, despite significant control efforts, FMD persists, with periodic outbreaks of serotypes A, O, and Asia 1 [8, 9]. The country's location, bridging Asia (where the disease is endemic) and Europe (which is disease-free), and the increased livestock movement in the region, make it a critical area for FMD control [8, 10].

FMD strains in Türkiye have been genetically linked to those circulating in the Middle East, suggesting that most introductions originate from the east. Within Türkiye, east-west animal movements, between production zones in the east and consumption centers in the west, have been incriminated as a key factor in the introduction of new strains and their westward spread [8]. While the region of Thrace in Türkiye, which borders the EU, was classified as FMD-free with vaccination in 2010 [11], the region of Anatolia remained endemic as of 2025 [11, 12]. In Thrace, FMD control included culling suspected cases, banning live animal imports from Anatolia, and enforcing strict hygiene rules. In southeastern Anatolia, bordering endemic regions, risk-based surveillance was intensified. Large ruminants were vaccinated twice yearly nationwide, while small ruminants were vaccinated annually in Thrace and additionally in affected zones in Anatolia during outbreaks [12–14].

Despite the reduction in FMD distribution and increased efforts to turn Türkiye into a disease-free country, the emergence of serotype SAT 2 in 2023 marked the first recorded detection of this serotype in Türkiye, requiring changes to existing control strategies, including the vaccine deployed in the field. The disease's persistence in Türkiye and the introduction of new serotypes are influenced by factors like host abundance, long-distance animal trade, and meat production demands, which provide opportunities for outbreaks [8, 12]. Another factor that might pose a risk for the introduction of new serotypes and spread of disease throughout the country is the occurrence of cultural or religious festivities, such as the Kurban Bayramı, during which animal movement and trade increase in large numbers [12, 14, 15].

Given this context, the present study aims to analyze the temporal and spatial distributions of FMD in Türkiye, assess long-term trends and seasonal patterns, and examine the impact of event-driven FMD outbreaks such as serotype introductions and Kurban Bayramı festivities.

## 2. Materials and Methods

### 2.1. Dataset

The dataset used for this study involved the number of FMD-reported outbreaks (villages affected by FMD outbreaks) in domestic species in Türkiye between November 2005 and March 2025. The serotype characterization was also provided in the dataset, with serotypes O, A, Asia 1, and SAT 2 identified, together with untyped serotypes. The dataset contained no outbreak data for the years 2008 and 2009.

### 2.2. Statistical Analysis

Descriptive statistics were employed to visualize the FMD-reported outbreaks across the country overtime. This was achieved using a combination of line charts, bar charts, and geographic maps. Maps were developed using shapefiles of the world from the public domain repository of Natural Earth [16]. Additionally, time series analysis and permutation tests [17] were also employed. All the analysis was done using the R Statistical Software (v4.4.0; R Core Team 2024).

### 2.3. Time Series Modeling

A time-series decomposition approach was applied to evaluate long-term trends (Tt), seasonal effects (St), and random fluctuations (Rt) in monthly FMD outbreak reports from 2016 to 2025. An additive decomposition model was selected after comparing additive and multiplicative structures [18]. Visual inspection indicated that the magnitude of seasonal fluctuations remained relatively constant throughout the study period, consistent with an additive structure. The St represents recurrent patterns within a time series, the Tt represents the long-term trend direction of a time series, and the Rt represents irregular fluctuations not explained by seasonality or trend. The package used for the time series analysis was the stats package [19].

### 2.4. Event-Driven Outbreaks

#### 2.4.1. Serotype Introduction

**Figure 1:**
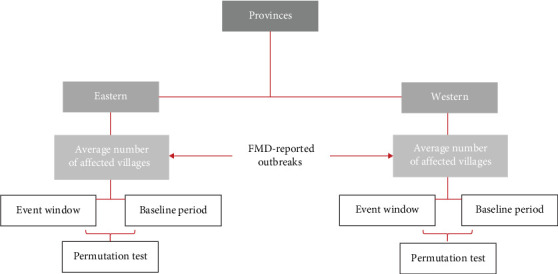
Nonparametric permutation test diagram. The permutation test was used to compare the average number of FMD-affected villages between the serotype introduction and Kurban Bayramı window and the baseline period.

The analysis included distinct serotypes, topotypes, lineages, or sublineages introduced in Türkiye. Information on these introductions, including their detection dates and genetic classification, was obtained from the World Reference Laboratory for FMD (WRLFMD) [20], the reference center for FMD characterization, the authors did not perform sample collection and diagnostic analyses. The total number of villages and provinces affected throughout the year when the introduction was made was calculated. In addition, the circulation of the same serotypes introduced in Türkiye was examined in surrounding regions, including Eastern Europe, Northern Africa, and Wstern and Central Asia, based on publicly available information from the WRLFMD. These data were used only to identify the timing and presence of serotype introductions in neighboring countries, as outbreak-level temporal data from these regions were not publicly available, preventing a direct comparison of FMD trends across countries. The provinces were divided into eastern and western provinces according to longitude. Provinces east of longitude 33° were considered eastern, and provinces west of longitude 33° were considered western. The average number of FMD-reported outbreaks was calculated, and nonparametric permutation tests were applied to assess whether changes between the number of outbreaks for the serotype introduction window and the baseline periods (i.e., average of reported outbreaks in the months outside the serotype introduction window) were significantly different for both eastern and western provinces (Figure 1). For each comparison, 50,000 random permutations were performed to approximate the null distribution of mean differences. Statistical significance was evaluated at the *α* = 0.05 level.

#### 2.4.2. Kurban Bayramı

The analysis included 15 Kurban Bayramı events between 2005 and 2025. Similar to what was done for serotype introductions, the provinces were divided into eastern and western according to longitude. The average number of FMD-reported outbreaks affected was calculated, and nonparametric permutation tests were applied to assess whether changes in the number of outbreaks between the Kurban Bayramı and baseline periods (i.e., the average of reported outbreaks in the months outside the Kurban Bayramı window) were significantly different for both eastern and western provinces (Figure 1). For each comparison, 50,000 random permutations were performed to approximate the null distribution of mean differences. Statistical significance was evaluated at the *α* = 0.05 level.

## 3. Results

### 3.1. Descriptive Analysis

**Figure 2:**
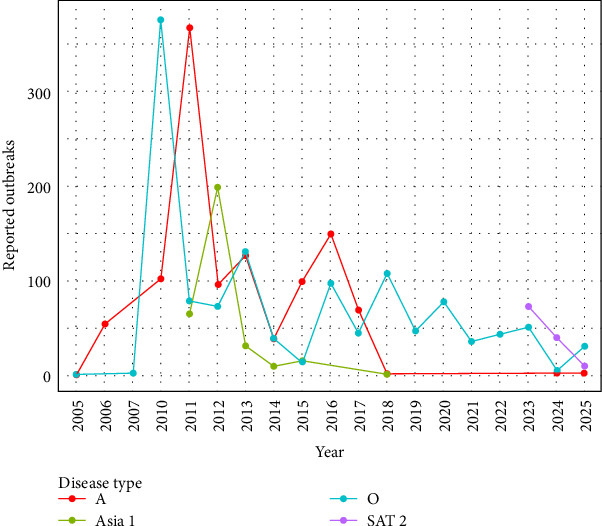
Count of FMD-reported outbreaks per year for each serotype: serotype A (orange line), serotype Asia 1 (green line), serotype O (blue line), and serotype SAT 2 (purple line).

**Figure 3:**
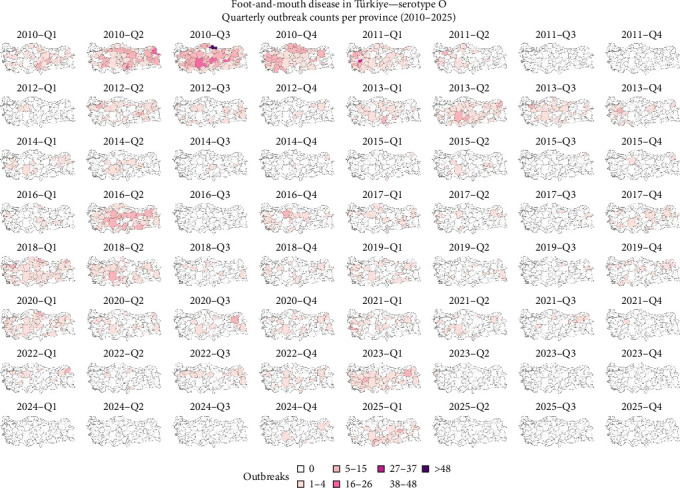
Spatial distribution of FMD-reported outbreaks caused by a serotype O virus in Türkiye between 2010 and 2025. Each panel represents the spatial distribution of reported outbreaks for a specific quarter. Provinces are shaded according to the number of reported outbreaks, with darker shades indicating higher outbreak intensity. Data source: National Surveillance Database and FAO EuFMD (Natural Earth base map).

**Figure 4:**
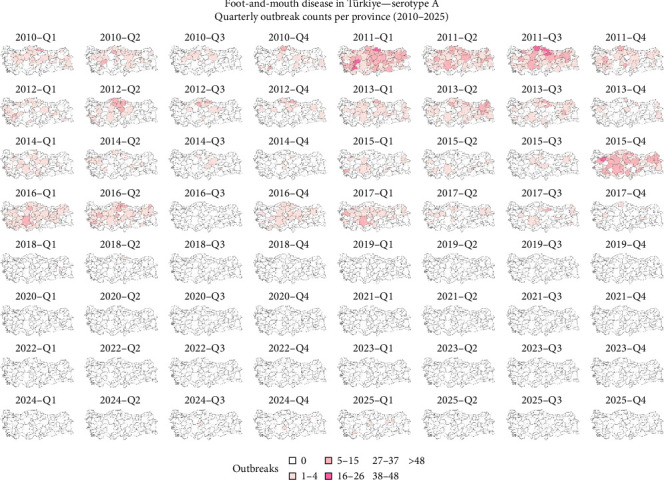
Spatial distribution of FMD-reported outbreaks caused by a serotype A virus in Türkiye between 2010 and 2025. Each panel represents the spatial distribution of reported outbreaks for a specific quarter. Provinces are shaded according to the number of reported outbreaks, with darker shades indicating higher outbreak intensity. Data source: National Surveillance Database and FAO EuFMD (Natural Earth base map).

**Figure 5:**
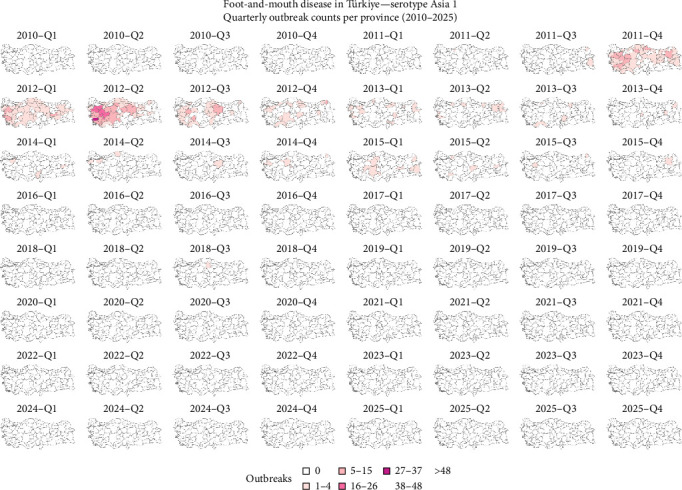
Spatial distribution of FMD-reported outbreaks caused by a serotype Asia-1 virus in Türkiye between 2010 and 2025. Each panel represents the spatial distribution of reported outbreaks for a specific quarter. Provinces are shaded according to the number of reported outbreaks, with darker shades indicating higher outbreak intensity. Data source: National Surveillance Database and FAO EuFMD (natural earth base map).

**Figure 6:**
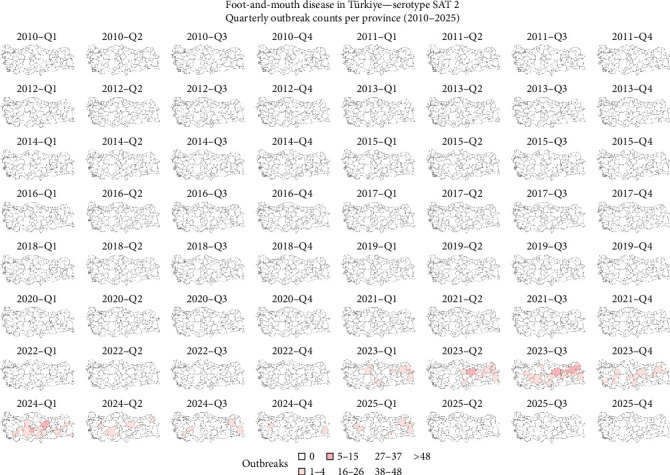
Spatial distribution of FMD-reported outbreaks caused by a serotype SAT 2 virus in Türkiye between 2010 and 2025. Each panel represents the spatial distribution of reported outbreaks for a specific quarter. Provinces are shaded according to the number of reported outbreaks, with darker shades indicating higher outbreak intensity. Data source: National Surveillance Database and FAO EuFMD (natural earth base map).

It was observed that the reported outbreaks per year reached their peak between 2010 and 2011, and from those years forward, the number of outbreaks has been decreasing (Figure 2). The geographical distribution of FMD-reported outbreaks revealed distinct temporal and spatial patterns across serotypes. Between 2010 and 2016, serotypes A, Asia 1, and O were widely distributed throughout the country. For serotype O, a decrease in outbreaks was observed between 2014 and 2015, followed by a surge in 2016. Although the number of outbreaks declined after 2016, reports continued to emerge from across the country, and between 2024 and March 2025, there was an increase in the number of outbreaks (Figure 3). Serotype A experienced a sharp decline in reported cases between 2017 and 2018 and reappeared in 2024 (Figure 4). Concerning serotype Asia 1, this serotype saw a reduction after 2015, without reported outbreaks until a single reemergence in 2018 (Figure 5). Serotype SAT 2 appeared in March 2023, with several outbreaks in the country, with a subsequent decline in the following months (Figure 6). Concerning the number of reported FMD outbreaks per season between 2005 and 2025, the highest number of outbreaks occurred in the spring, with an increased number of outbreaks between February and May.

### 3.2. Time Series Modeling

**Figure 7:**
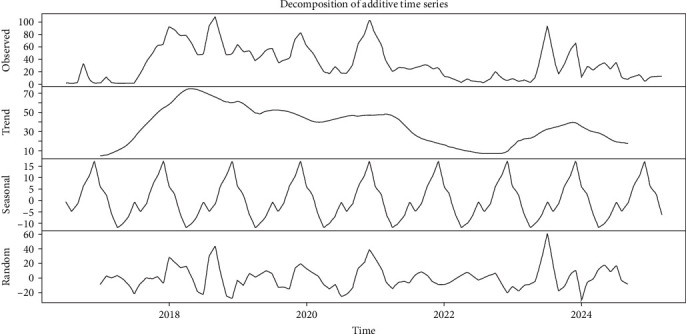
Time series for the number of reported outbreaks of foot-and-mouth disease between 2016 to 2025.

The time series of FMD-reported outbreaks between 2016 and 2025 revealed a seasonal structure, along with clear long-term trends and irregular components (irregular fluctuations not explained by seasonality or trend). The seasonal component displayed peaks in the first quarter of the year, an occurrence that seems consistent throughout the years. A decreasing trend was observed between 2016 and 2025, with a slight increase in 2023 coinciding with the introduction of SAT 2 in Türkiye (Figure 7). The random component captured residual variation, particularly during outbreak years. Notably, in 2023, the residuals had a positive peak, indicating that in this year the number of FMD-reported outbreaks was above what would be expected based on trend and seasonality alone (Figure 7).

### 3.3. Event-Driven Outbreaks: Serotype Introduction

In 2010, two different sublineages (BR-08 and AFG-07) of serotype A and three different sublineages (SAN-09, FAR-09 and ANT-10) of serotype O were observed. In 2011, a new sublineage (SIS-10) of serotype A was introduced, along with the introduction of serotype Asia 1. In 2015, another introduction of a different lineage (G-VII) of serotype A was noted. In 2017, a distinct sublineage (QOM-15) of serotype O was observed. Finally, in 2023, the introduction of serotype SAT 2 was documented. The serotype introductions over the years revealed a big spike in FMD-reported outbreaks between 2010 and 2011, with a total of 76 and 77 out of 81 provinces affected in those years, respectively. Additionally, it was noted that the number of provinces affected has been decreasing over time. This trend occurred even after the introduction of serotype SAT 2, which led to an increase in the number of reported outbreaks but affected fewer provinces compared to previous years. Furthermore, it was observed that for most serotype introductions, the same serotype was circulating in countries neighboring Türkiye during the year of its introduction in Türkiye or in the year prior (Table 1).

**Figure 8:**
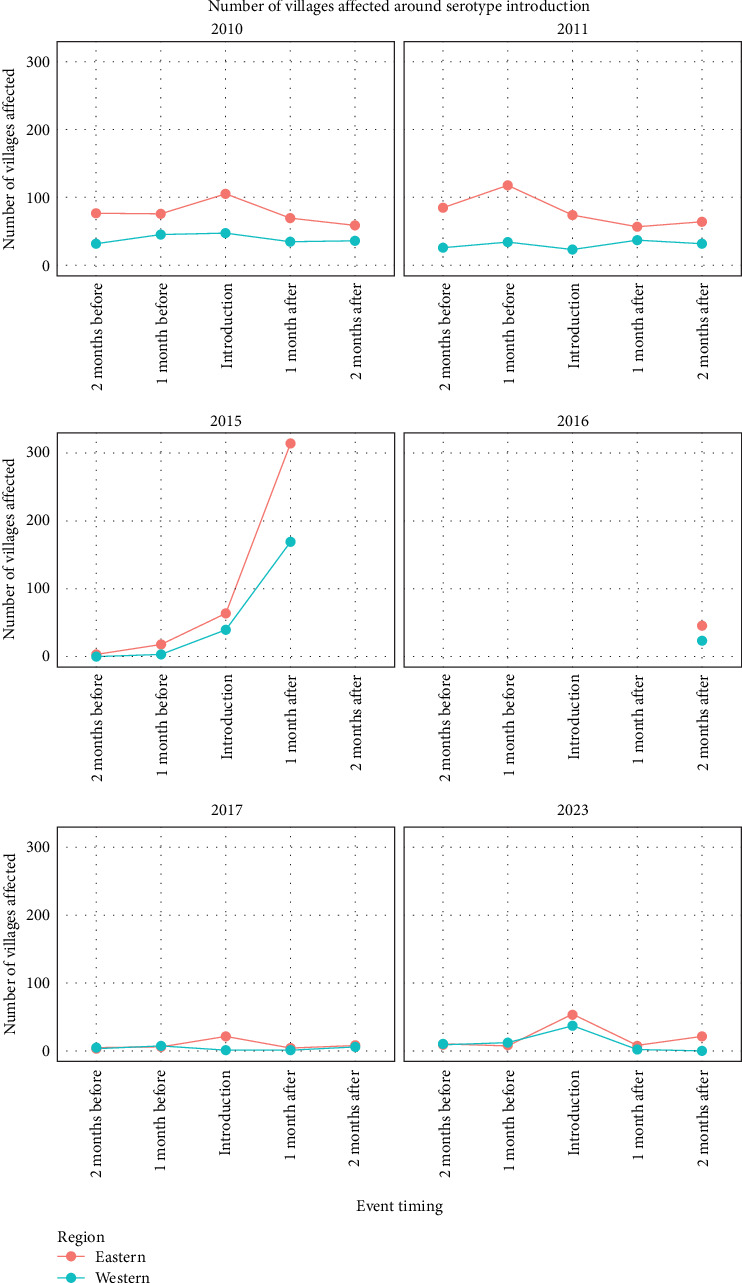
FMD-reported outbreaks for the eastern (orange line) and western (blue line) provinces affected in the months preceding and following the serotype introduction.

It was observed that overall, eastern provinces had a higher number of reported outbreaks (villages affected by FMD) than western provinces in the months surrounding the serotype introductions (Figure 8). It was also observed that the difference between the number of FMD-reported outbreaks was higher in the months surrounding the serotype introduction compared to the months outside the serotype introduction window, as shown by the permutation test, with a significant difference for both eastern (*p*-value 0.03) and western provinces (*p*-value 0.02, Table 2).

### 3.4. Event-Driven Outbreaks: Kurban Bayramı

**Figure 9:**
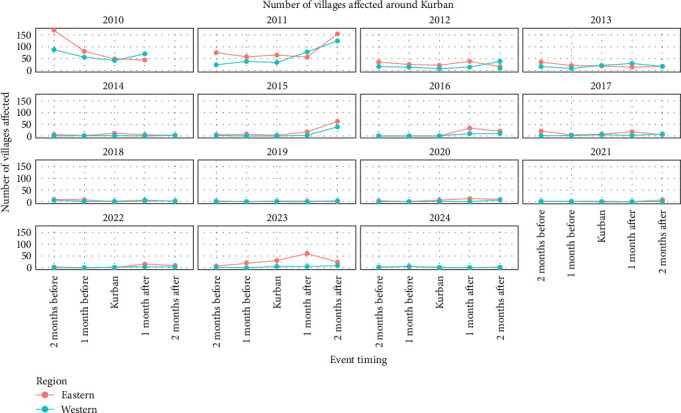
FMD-reported outbreaks for the eastern (orange line) and western (blue line) provinces affected in the months preceding and following the Kurban Bayramı.

Regarding Kurban Bayramı festivities in Türkiye, it was observed that overall eastern provinces had a higher number of reported outbreaks (villages affected by FMD) than western provinces in the months surrounding the festivities (Figure 9). It was also observed that the difference between the number of FMD-reported outbreaks was higher in the months surrounding the Kurban Bayramı compared to the months outside the Kurban Bayramı window, as shown by the permutation test, with a significant difference for eastern provinces (*p*-value 0.02) and a marginally significant difference for western provinces (*p*-value 0.06, Table 3).

## 4. Discussion

This study examined the temporal trends, serotype dynamics, and seasonal patterns of FMD in Türkiye. The findings indicated that FMD-reported outbreaks demonstrated seasonality, with peaks during the spring months and a decreasing trend across the years. Furthermore, the introduction of serotypes in Türkiye was preceded by the circulation of these serotypes in neighboring countries during the same year or the year prior and was followed by an increase in the number of affected villages in Türkiye. Additionally, it was also observed that the number of villages affected by FMD increased in the months surrounding the Kurban Bayramı festivities.

Türkiye's unique geographic position, as a transcontinental bridge between Asia and Europe, places it at the heart of regional FMD transmission dynamics. As described [8, 10, 21], the country lies along a livestock corridor connecting endemic and FMD-free regions. Livestock trade, especially from eastern production zones toward western markets, is a known driver of disease dissemination [22]. Historically, Türkiye has been endemic for serotypes O and A, with intermittent circulation of Asia 1 [8, 23]. Among these, serotype O has consistently demonstrated the highest prevalence and impact. Its elevated transmissibility and adaptability across species contribute to its persistence and widespread outbreaks [8, 24]. This aligns with the findings in this study, where serotype O was associated with a high number of FMD outbreaks and persistence in the country across the years. In contrast, serotype A, while historically present, showed lower persistence, potentially due to reduced host susceptibility or more effective containment [2, 8]. That was also observed in the results from this study, where a peak of the number of FMD outbreaks was observed between 2010 and 2011, decreasing abruptly to much lower values that remained low until 2025, when it resurged again. Concerning serotype Asia 1, its sporadic nature further supports its classification as a transient serotype in the region [25]. This study also showed that the number of outbreaks due to serotype Asia 1 was lower than the number of outbreaks due to serotypes O and A, and similar to what happened with serotype A, FMD outbreaks due to serotype Asia 1 reached a peak in 2012 and decreased in the following years, with no reported outbreaks after 2018. The elevated number of reported outbreaks between 2007 and 2013; however, may not solely reflect increased viral activity but could also be partly explained by enhanced diagnostic capacity and improved disease reporting systems established during this period, since Türkiye initiated major FMD control and surveillance [26].

Regarding seasonality, FMD outbreaks peaked during the spring months. This pattern may be explained by seasonal grazing practices and increased livestock movement, as well as a higher proportion of susceptible young animals entering the population. Similar seasonal trends have been documented in other endemic countries [27–29], emphasizing the importance of synchronizing control measures, particularly vaccination campaigns, with periods of heightened risk.

An increased live animal movement is known to take place in the months preceding and following the Kurban Bayramı festivities in Türkiye, which can be a factor contributing to disease spread [30]. In this study, it was observed that overall, the number of villages affected by FMD increased in the months surrounding the Kurban Bayramı festivities, with eastern provinces showing a higher number of reported outbreaks than western provinces. By analyzing the line charts (Figure 9), it can be observed that the years 2010, 2011, and 2023 were the years with a higher number of villages affected in the months surrounding the Kurban Bayramı, which were years where new serotype introductions were reported. Although contingency plans and enhanced biosecurity measures have been considered and implemented during the festivities, increases in the number of outbreaks or the introduction of new serotypes can still occur. This can be due to the surge in informal and unregulated animal and human movements driven by high demand during the festivities [31]. In light of these challenges, strengthening community-based disease surveillance and improving communication between veterinary authorities and livestock owners before animal movements may help reduce the risk of transporting infected animals and prevent future outbreaks.

Accurate identification and characterization of FMD serotypes, lineages, and sublineages are essential, as vaccines offer no cross-protection between serotypes, and new serotypes can significantly affect livestock production in naïve populations. Additionally, as viral types are often geographically restricted, their identification is important for tracing the origins of outbreaks and understanding the distribution and spread of the disease across the country [32], thereby contributing to national disease control efforts. In this study, it was observed that new serotype introductions in Türkiye were happening in the year or in the year after that same serotype was circulating in surrounding countries [31, 32], which emphasizes the need for enhanced disease surveillance when animal movements occur between countries [21, 33]. It was also observed that even though new introductions were still happening, the number of provinces affected was smaller compared to those affected between 2010 and 2016. The faster time to control the spread between provinces was likely due to strengthened vaccination efforts, improved diagnostics, and more rigorous control strategies. Concerning vaccination, Türkiye implements a biannual mass vaccination program for cattle, including primary and booster doses, to maintain herd immunity and control FMD outbreaks [34]. As part of its national program, Türkiye set a goal to achieve FMD-free status without vaccination in the Thrace region and with vaccination in Anatolia. This aligns with reports of enhanced vaccine production and administration [23] and with the development of training tools for veterinarians from the Turkish General Directorate of Food and Control [13] and the Progressive Control Pathway-FMD (PCP-FMD) [35]. However, detailed national-level vaccine coverage data were not publicly available, limiting quantitative assessment of the direct impact of vaccination on outbreak trends in this study. Future studies that integrate serosurveillance and seromonitoring datasets from national or regional authorities would provide valuable insight into how outbreak dynamics relate to vaccine effectiveness and population immunity.

The analysis revealed significant differences between eastern and western provinces in the impact of serotype introductions and the Kurban period on FMD outbreaks. While specific data on regional veterinary infrastructure or surveillance intensity were not available for this study, previous reports provide some context. Spatiotemporal analyses of FMD in Türkiye between 2010 and 2019 indicated higher case numbers in eastern and southeastern provinces, potentially due to more intensive animal movements and proximity to international borders [9]. Ongoing efforts to improve clinical surveillance and veterinary infrastructure in these border regions were also reported, suggesting that disparities in resources and access may contribute to the observed patterns [36, 37]. However, without direct comparative data on surveillance intensity or veterinary capacity, the influence of these regional factors on outbreak patterns remains uncertain and warrants further investigation.

Despite significant progress, several limitations must be acknowledged. For the analysis section focused on event-driven outbreaks, only Kurban Bayramı and serotype introductions were considered, and other factors that were not analyzed in this study might also have had an impact on disease spreading. These include socioeconomic factors, social unrest in neighboring countries, livestock market dynamics, environmental conditions, or changes in control policies. Misclassification of serotypes and underreporting of outbreaks may also have affected the accuracy of the findings. Studies have shown that underreporting is a common issue in regions with limited health infrastructures, political instability, or logistical challenges [38]. Conflict zones and remote areas, in particular, often experience disruptions to veterinary services, delays in outbreak detection, and limited communication networks, which can result in outbreaks going unreported and an underestimation of disease spread [38, 39]. With that in mind, two factors that might have influenced FMD spread or new introductions were the civil war in Syria that started in 2011, which led to a wave of refugees into Türkiye, and the earthquake in 2023 [40]. Similarly, in remote areas with limited access to communication networks, outbreaks may go unreported, skewing the data and potentially underestimating the true extent of disease spread [41].

Future research should aim to integrate detailed information on surveillance intensity, vaccine coverage, and animal movement controls into epidemiological models. Understanding how these factors interact with environmental and socioeconomic drivers will be crucial for designing adaptive and cost-effective FMD control strategies. Accounting for seasonal dynamics can further optimize the timing of vaccination and surveillance programs, particularly during peak-risk periods such as spring. Biological factors, including serotype-specific immune responses and vaccine-induced immunity, may also contribute to the observed differences in outbreak patterns. Future studies incorporating serological or vaccine efficacy data could help clarify these interactions. Additionally, linking outbreak trend analyses with real-time early warning systems, predictive models, and regional data sharing could enhance timely detection and coordinated control of FMD across Türkiye and neighboring regions.

## 5. Conclusion

This study provided a comprehensive analysis of the temporal trends, seasonal patterns, and serotype dynamics of FMD in Türkiye from 2005 to 2025. The findings highlighted clear seasonality in FMD outbreaks, with peaks in the spring months. Serotype O remained the most dominant and impactful throughout the study period, associated with the highest number of outbreaks. Despite overall declines in FMD reported outbreaks in recent years, the persistence and evolution of the disease, driven by the introduction of new serotypes and possibly underreported or untyped strains, underscore the need for sustained and adaptive control efforts. Investment in serotype-specific surveillance, timely diagnostics, and targeted vaccination strategies are essential, particularly considering Türkiye's aim to achieve FMD disease-free status. Strengthening regional cooperation and tailoring control measures to account for seasonal patterns can be the key to reducing FMD's impact and moving closer toward long-term disease control.

## Figures and Tables

**Table 1:** Dynamics of FMD introductions in Türkiye per serotype, topotype, lineage, and sublineage.

Date	Serotype	Topotype	Lineage	Sublineage	# of provinces affected (out of 81)	Neighboring countries reporting the same serotype (year)
4/2/2010	A	ASIA	Iran-05	BAR-08	76	Libya, Iraq, and Iran (2009)
2/9/2010	O	ME-SA	PanAsia	SAN-09		.
2/9/2010	O	ME-SA	PanAsia	FAR-09		Iran (2009)
2/9/2010	O	ME-SA	PanAsia	ANT-10		Iran (2010)
2/9/2010	A	ASIA	Iran-05	AFG-07		Iran (2009)

29/9/2011	A	ASIA	Iran-05	SIS-10	77	Iran (2010)
29/9/2011	Asia 1	ASIA	Sindh-08	—		Iran (2011)

9/11/2015	A	ASIA	G-VII	—	68	Armenia and Iran (2015)

14/7/2017	O	ME-SA	PanAsia-2	QOM-15	63	Iran (2016)

16/3/2023	SAT 2	XIV	—	—	50	Iraq (2023)

*Note:* The table illustrates the number of provinces affected and the circulation of the same serotype in countries surrounding Türkiye.

**Table 2:** Permutation test results for the serotype introduction in eastern and western provinces.

Province	Serotype introduction (average villages affected)	Baseline (average villages affected)	Difference	*p*-Value
Eastern provinces: serotype introduction vs. baseline	265	41	224	0.03
Western provinces: serotype introduction vs. baseline	127	19	108	0.02

*Note:* The table shows the difference in the number of villages affected during the serotype introduction period compared to the baseline (average of the number of villages affected outside of the serotype introduction period).

**Table 3:** Permutation test results for the Kurban Bayramı in eastern and western provinces.

Province	Kurban (average villages affected)	Baseline (average villages affected)	Difference	*p*-Value
Eastern provinces: Kurban vs. baseline	99	28	71	0.02
Western provinces: Kurban vs. baseline	60	17	43	0.06

*Note:* The table shows the difference in the number of villages affected during the Kurban Bayramı period compared to the baseline (average of the number of villages affected outside of the Kurban Bayramı period).

## Data Availability

The data that support the findings on the number of foot-and-mouth disease number of outbreaks are available from the Food and Agriculture Organization of the United Nations. Restrictions apply to the availability of these data, which were used under license for this study. Data are available with the permission of the Food and Agriculture Organization of the United Nations. The data concerning the serotype introductions is available at https://www.wrlfmd.org/.

## References

[1] Grubman M. J., Baxt B. (2004). Foot-and-Mouth Disease. *Clinical Microbiology Reviews*.

[2] Elrashedy A., Nayel M., Salama A., Zaghawa A., El-Shabasy R. M., Hasan M. E. (2025). Foot-and-Mouth Disease: Genomic and Proteomic Structure, Antigenic Sites, Serotype Relationships, Immune Evasion, Recent Vaccine Development Strategies, and Future Perspectives. *Veterinary Research*.

[3] Domingo E., Baranowski E., Escarmís C., Sobrino F. (2002). Foot-and-Mouth Disease Virus. *Comparative Immunology, Microbiology and Infectious Diseases*.

[4] Jemberu W. T., Mourits M. C. M., Woldehanna T., Hogeveen H. (2014). Economic Impact of Foot and Mouth Disease Outbreaks on Smallholder Farmers in Ethiopia. *Preventive Veterinary Medicine*.

[5] Wong C. L., Yong C. Y., Ong H. K., Ho K. L., Tan W. S. (2020). Advances in the Diagnosis of Foot-and-Mouth Disease. *Frontiers in Veterinary Science*.

[6] Blake A., Sinclair M. T., Sugiyarto G. (2001). The Economy-Wide Effects of Foot and Mouth Disease in the UK Economy. *Christel DeHaan Tourism and Travel Research Institute*.

[7] Hsu S.-H., Lee D.-H., Chang C.-C., Lin H.-C., Yang T.-C. (2005). An Ex Post Evaluation of Economic Impacts of Foot-and-Mouth Disease on Taiwan Using a Dynamic Computable General Equilibrium Model. *AgEcon*.

[8] Gilbert M., Aktas S., Mohammed H. (2005). Patterns of Spread and Persistence of Foot-and-Mouth Disease Types A, O and Asia-1 in Turkey: A Meta-Population Approach. *Epidemiology and Infection*.

[9] Bayir T., Gürcan İ. S. (2022). Spatiotemporal Distributions of Foot and Mouth Disease Between 2010–2019 in Turkey. *Acta Veterinaria*.

[10] Herrera-Diestra J. L., Tildesley M., Shea K., Ferrari M. J. (2022). Cattle Transport Network Predicts Endemic and Epidemic Foot-and-Mouth Disease Risk on Farms in Turkey. *PLOS Computational Biology*.

[11] World Organisation for Animal Health (WOAH) (2025). The State of the World’s Animal Health. https://www.woah.org/app/uploads/2025/05/the-state-of-the-worlds-animal-health-2025.pdf.

[12] Arede M., Beltrán-Alcrudo D., Aliyev J. (2023). Examination of Critical Factors Influencing Ruminant Disease Dynamics in the Black Sea Basin. *Frontiers in Veterinary Science*.

[13] European Commission for the Control of Foot-and-Mouth Disease (EuFMD) (2021). Online Course: FITC in Turkey. https://www.fao.org/eufmd/meetings-and-events/detail/en/c/1402023/.

[14] Food and Agriculture Organization of the United Nations (FAO) (2024). Foot-and-Mouth Disease Quarterly Report.

[15] Tuppurainen E., Oura C. (2014). Lumpy Skin Disease: An African Cattle Disease Getting Closer to the EU. *Veterinary Record*.

[16] Natural Earth (2025). Natural Earth Data: Public Domain Map Dataset. http://www.naturalearthdata.com/.

[17] Good P. (2000). *Permutation Tests*.

[18] Hyndman R., Khandakar Y. (2008). Automatic Time Series Forecasting: The Forecast Package for R. *Journal of Statistical Software*.

[19] R Core Team (2013). *R: A Language and Environment for Statistical Computing*.

[20] World Reference Laboratory for Foot-and-Mouth Disease (WRLFMD) (2025). https://www.wrlfmd.org/western-and-central-asia/turkiye.

[21] Aslam M., Alkheraije K. A. (2023). The Prevalence of Foot-and-Mouth Disease in Asia. *Frontiers in Veterinary Science*.

[22] Knight-Jones T. J. D., Rushton J. (2013). The Economic Impacts of Foot and Mouth Disease: What are They, How Big are They and Where do They Occur?. *Preventive Veterinary Medicine*.

[23] Food and Agriculture Organization of the United Nations (FAO) (2018). Current FMD Situation, Control Strategy in Turkey & Perspectives for the Better Control of FMD in West Eurasia. *95th Executive Committee*. https://www.fao.org/fileadmin/user_upload/eufmd/Executive_committee95/Appendix_14_Turkey_report__N.Bulut_.pdf.

[24] Mardones F., Perez A., Sanchez J., Alkhamis M., Carpenter T. (2010). Parameterization of the Duration of Infection Stages of Serotype O Foot-and-Mouth Disease Virus: An Analytical Review and Meta-Analysis With Application to Simulation Models. *Veterinary Research*.

[25] Khounsy S., Conlan J. V., Gleeson L. J. (2009). Molecular Epidemiology of Foot-and-Mouth Disease Viruses From South East Asia 1998–2006: The Lao Perspective. *Veterinary Microbiology*.

[26] European Commission (EU EC) (2010). Standard Summary Project Fiche — IPA Decentralised National Programmes. https://enlargement.ec.europa.eu/document/download/e3ec3268-b9ee-4bd9-8f48-53170cf0a59b_en?filename=131_tr2010.0136.01_strategic_management_capacity.pdf.

[27] Ahmed H. A., Salem S. A. H., Habashi A. R. (2012). Emergence of Foot-and-Mouth Disease Virus SAT 2 in Egypt During 2012. *Transboundary and Emerging Diseases*.

[28] Kandeil A., El-Shesheny R., Kayali G. (2013). Characterization of the Recent Outbreak of Foot-and-Mouth Disease Virus Serotype SAT2 in Egypt. *Archives of Virology*.

[29] Giasuddin M., Ali M. Z., Sayeed M. A., Islam E., Mahmud M. S. (2020). Prevalence of Foot and Mouth Disease (FMD) in Different Affected Regions of Bangladesh and Its Economic Losses. *Bangladesh Journal of Livestock Research*.

[30] Arede M. (2024). Assessment and Risk Mapping of Ruminant Diseases in the Black Sea Basin. *Universitat Autònoma de Barcelona*.

[31] De Clercq K., Cetre-Sossah C., Métras R. (2018). Mission of the Community Veterinary Emergency Team to Bulgaria (16–21 July 2018). *European Commission*.

[32] Foglia E. A., Lembo T., Kazwala R. (2021). Combining Multiple Assays Improves Detection and Serotyping of Foot-and-Mouth Disease Virus: A Practical Example With Field Samples From East Africa. *Viruses*.

[33] Di Nardo A., Knowles N. J., Paton D. J. (2011). Combining Livestock Trade Patterns With Phylogenetics to Help Understand the Spread of Foot and Mouth Disease in Sub-Saharan Africa, the Middle East and Southeast Asia. *Revue Scientifique et Technique de l’OIE*.

[34] Knight-Jones T. J. D., Gubbins S., Bulut A. N. (2016). Mass Vaccination, Immunity and Coverage: Modelling Population Protection Against Foot-and-Mouth Disease in Turkish Cattle. *Scientific Reports*.

[35] Food and Agriculture Organization of the United Nations (FAO), GF-TADS, EuFMD (2018). The Progressive Control Pathway for Foot and Mouth Disease Control (PCP-FMD). https://openknowledge.fao.org/server/api/core/bitstreams/117c6cd3-3a59-473f-82a9-19390271b610/content.

[36] Yazicioğlu N. (2019). West Eurasia Roadmap for Control of FMD – Turkey. GF-TADS meeting. https://rr-europe.woah.org/app/uploads/2019/11/10_gf-tads-rsc5_s4_fmd-westeurasia-roadmap.pdf.

[37] European Commission for the Control of Foot-and-Mouth Disease (EuFMD), FAO, OIE (2013). *Tripartite Meeting on Control of FMD and Other Exotic Diseases in the Southern Balkans*.

[38] Marou V., Vardavas C. I., Aslanoglou K. (2024). The Impact of Conflict on Infectious Disease: A Systematic Literature Review. *Conflict and Health*.

[39] Tedla M. G., Berhe K. F., Grmay K. M. (2023). The Impact of Armed Conflict on Animal Well-Being and Welfare: The Case of Ethiopia’s Tigray Region. *Heliyon*.

[40] Department for Environment, Food and Rural Affairs (DEFRA) (2023). *Foot and Mouth Disease in the Middle East and Türkiye*.

[41] Worsley-Tonks K. E. L., Bender J. B., Deem S. L. (2022). Strengthening Global Health Security by Improving Disease Surveillance in Remote Rural Areas of Low-Income and Middle-Income Countries. *The Lancet Global Health*.

